# Supplementary material for: JMJD6 is a tumorigenic factor and therapeutic target in neuroblastoma
Source: Nat Commun. 2019 Jul 25;10:3319. doi: 10.1038/s41467-019-11132-w (PMC6658504; doi:10.1038/s41467-019-11132-w)
Supplement: Supplementary file 1 — Supplementary Information [file 41467_2019_11132_MOESM1_ESM.pdf]

Supplementary Information

JMJD6 is a tumorigenic factor and therapeutic target in neuroblastoma

Wong et al

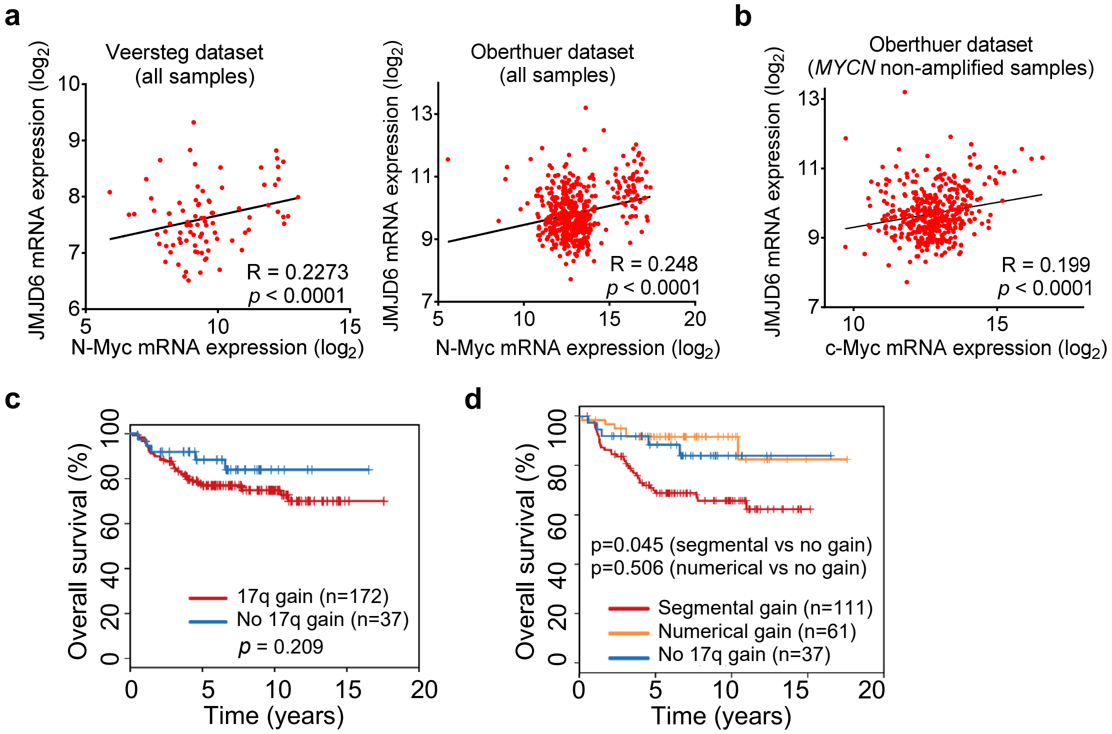

Supplementary Fig. 1. JMJD6 mRNA expression correlates with N-Myc and c-Myc mRNA expression in human neuroblastoma tissues, and only chromosome 17q segmental gain is associated with poor patient overall survival. **a, b** Two-sided Pearson's correlation was employed to analyze correlation between JMJD6 and N-Myc mRNA expression in the 88 and 476 human neuroblastoma tissues of the microarray gene expression Versteeg and Oberthuer datasets (**a**), or correlation between JMJD6 and c-Myc mRNA expression in the 405 *MYCN*-non-amplified human neuroblastoma tissues in the large Oberthuer dataset (**b**). **c, d** Kaplan–Meier curves showed the probability of overall survival of patients according to overall chromosome 17q gain (segmental and numerical gain) (**c**), segmental gain alone or numerical gain alone (**d**), in the 209 neuroblastoma samples with array-CGH data. Source data are provided as a Source Data file.

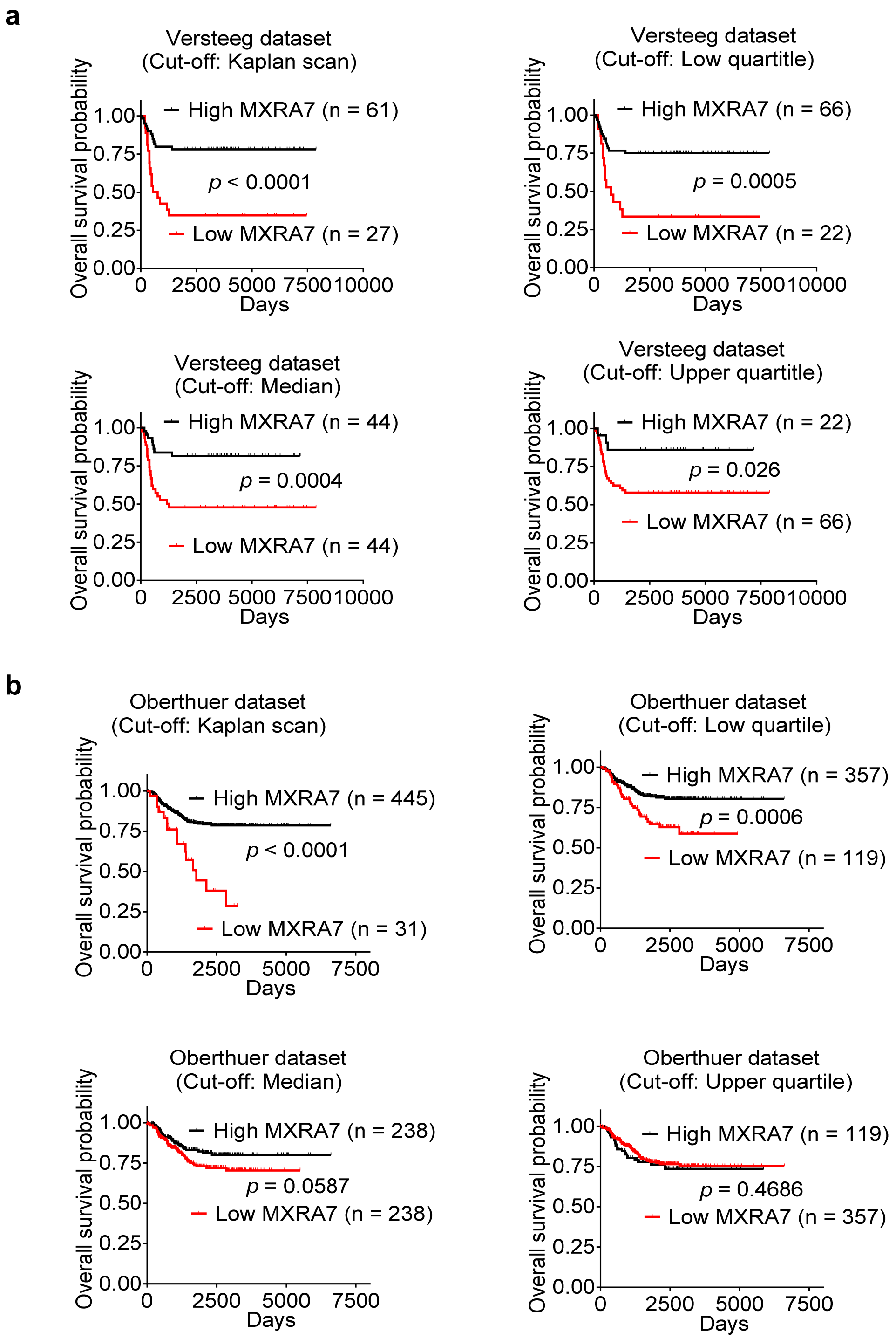

Supplementary Fig. 2. High levels of *MXRA7* gene expression in human neuroblastoma tissues do not correlate with poor patient prognosis. **a, b** Kaplan–Meier curves showed the probability of overall survival of patients according to MXRA7 mRNA expression levels in the 88 and 476 neuroblastoma samples in the Versteeg (**a**) and Oberthuer (**b**) datasets, using the optimal cut-off level determined by Kaplan scanning, the median, upper or low quartile of MXRA7 mRNA expression as the cut-off points. Source data are provided as a Source Data file.

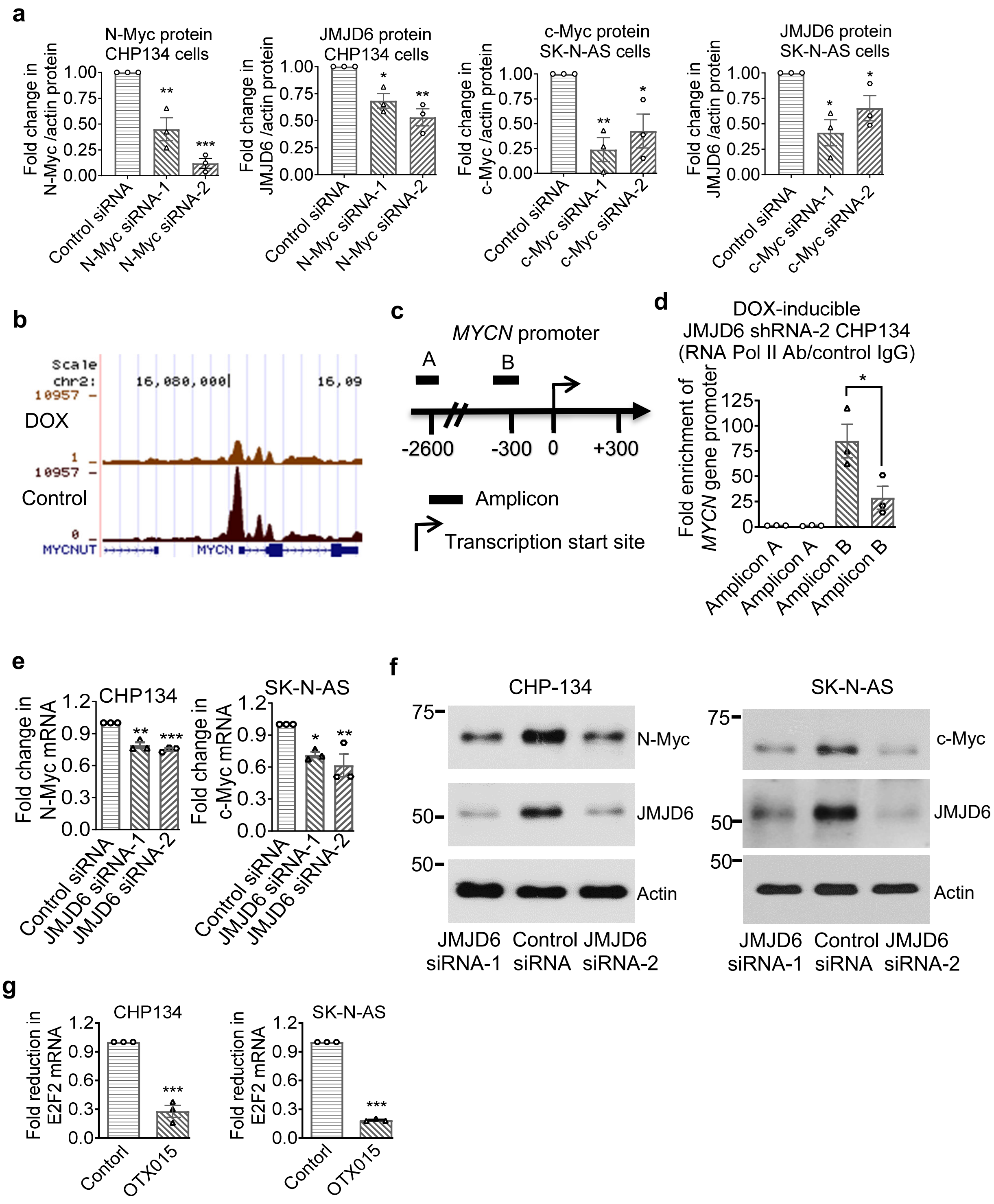
a
N-Myc protein CHP134 cells
Fold change in N-Myc /actin protein
Control siRNA
N-Myc siRNA-1
N-Myc siRNA-2
JMJD6 protein CHP134 cells
Fold change in JMJD6 /actin protein
c-Myc protein SK-N-AS cells
Fold change in c-Myc /actin protein
c-Myc siRNA-1
c-Myc siRNA-2
JMJD6 protein SK-N-AS cells
b
Scale
chr2:
16,080,000
DOX
Control
MYCNUT
MYCN
c
MYCN promoter
A
B
-2600
-300
0
+300
Amplicon
Transcription start site
d
DOX-inducible JMJD6 shRNA-2 CHP134 (RNA Pol II Ab/control IgG)
Fold enrichment of MYCN gene promoter
Amplicon A
Amplicon B
e
CHP134
Fold change in N-Myc mRNA
SK-N-AS
Fold change in c-Myc mRNA
JMJD6 siRNA-1
JMJD6 siRNA-2
f
CHP-134
SK-N-AS
N-Myc
c-Myc
JMJD6
Actin
JMJD6 siRNA-1
Control siRNA
JMJD6 siRNA-2
g
Fold reduction in E2F2 mRNA
Contorl
OTX015

Supplementary Fig. 3. Myc is important for JMJD6 expression and JMJD6 is importasnt for E2F2 and Myc expression. **a** CHP134 cells were transfected with control siRNA, N-Myc siRNA-1 or N-Myc siRNA-2, and SK-N-AS cells were transfected with control siRNA, c-Myc siRNA-1 or c-Myc siRNA-2. Forty-eight hours later, protein were extracted from the cells for immunoblot analyses of JMJD6, N-Myc and c-Myc protein expression. Representative immunoblot gels were shown in Fig. 2b. JMJD6, N-Myc and c-Myc protein expression was quantified, relative to actin protein expression. Error bars represent normalized standard errors from three independent experiments (* $p < 0.05$, ** $p < 0.01$, *** $p < 0.001$, one-way ANOVA). **b** ChIP sequencing was performed with an anti-RNA Pol II antibody in doxycycline (DOX)-inducible JMJD6 shRNA-2 cells after treatment with vehicle control or DOX for 48 hours. **c** Schematic representation of the *MYCN* gene promoter. **d** ChIP assays were performed with a control IgG or anti-RNA Pol II antibody (Ab), followed by PCR with primers targeting the negative control region (Amplicon A) or the *MYCN* gene promoter (Amplicon B) in CHP134 cells. Fold enrichment of the *MYCN* gene promoter was calculated as the difference in cycle thresholds obtained with the anti-RNA Pol II Ab and with the control IgG. Error bars represent normalized standard errors from three independent experiments (* $p < 0.05$, two-tailed unpaired Student's t-test). **e, f** CHP134 and SK-N-AS cells were transfected with control siRNA, JMJD6 siRNA-1 or JMJD6 siRNA-2 for 48 hours. RNA and protein were extracted from the cells, followed by RT-PCR analysis of N-Myc and c-Myc mRNA expression (**e**) and immunoblot analysis of N-Myc, c-Myc and JMJD6 protein expression (**f**). Error bars represent normalized standard errors from three independent experiments (* $p < 0.05$, ** $p < 0.01$, *** $p < 0.001$, one-way ANOVA). **g** CHP134 cells were treated with vehicle control or 1μM OTX015, and SK-N-AS cells were treated with vehicle control or 2μM OTX015, for 48 hours, followed by RNA extraction and RT-PCR analysis of E2F2. Error bars represent normalized standard errors from three independent experiments (*** $p < 0.01$, two-tailed unpaired Student's t-test). Source data are provided as a Source Data file.

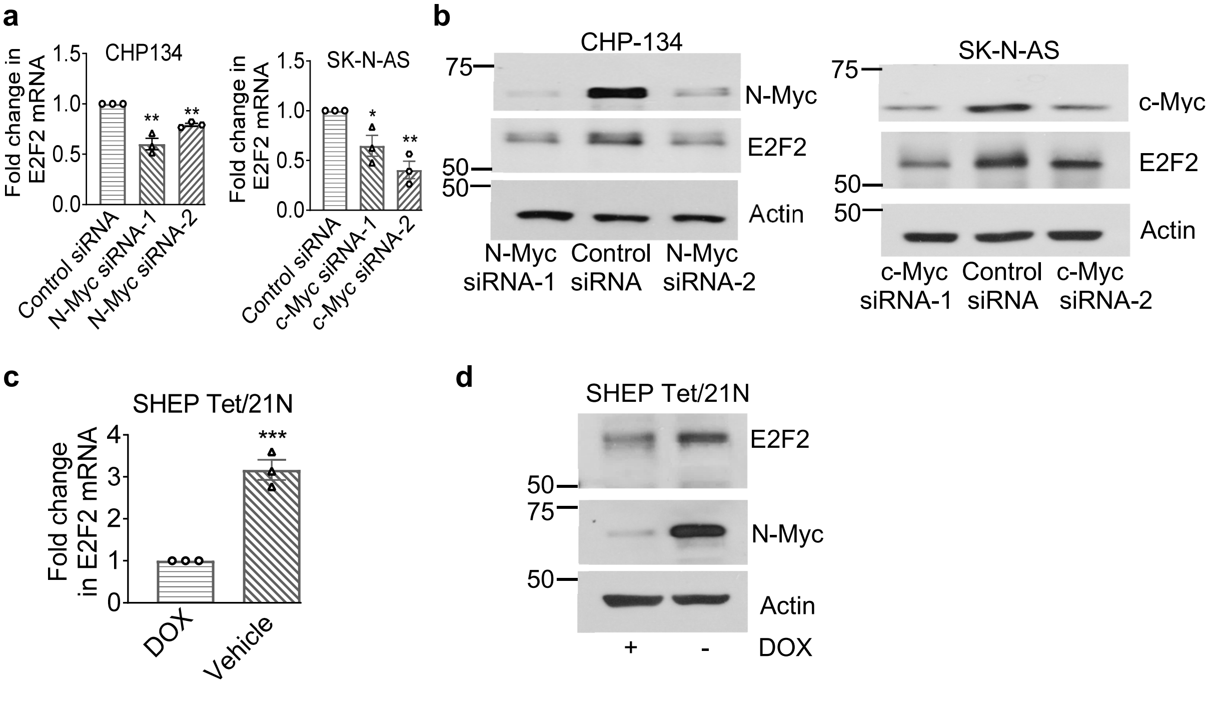

Supplementary Fig. 4. N-Myc and c-Myc up-regulate E2F2 expression. **a, b** CHP134 cells were transfected with control siRNA or N-Myc siRNAs, and SK-N-AS cells were transfected with control siRNA or c-Myc siRNAs. Forty-eight hours later, RNA and protein were extracted from the cells for RT-PCR (**a**) and immunoblot (**b**) analysis. **c, d** SHEP Tet/21N cells were treated with DOX (2µg/ml) or vehicle control for 48 hours. RT-PCR and immunoblot analyses of E2F2 and N-Myc were conducted. Error bars represent normalized standard errors from three independent experiments (* $p < 0.05$, ** $p < 0.01$, *** $p < 0.001$, two-tailed unpaired Student's t-test for two groups and one-way ANOVA for three groups). Source data are provided as a Source Data file.

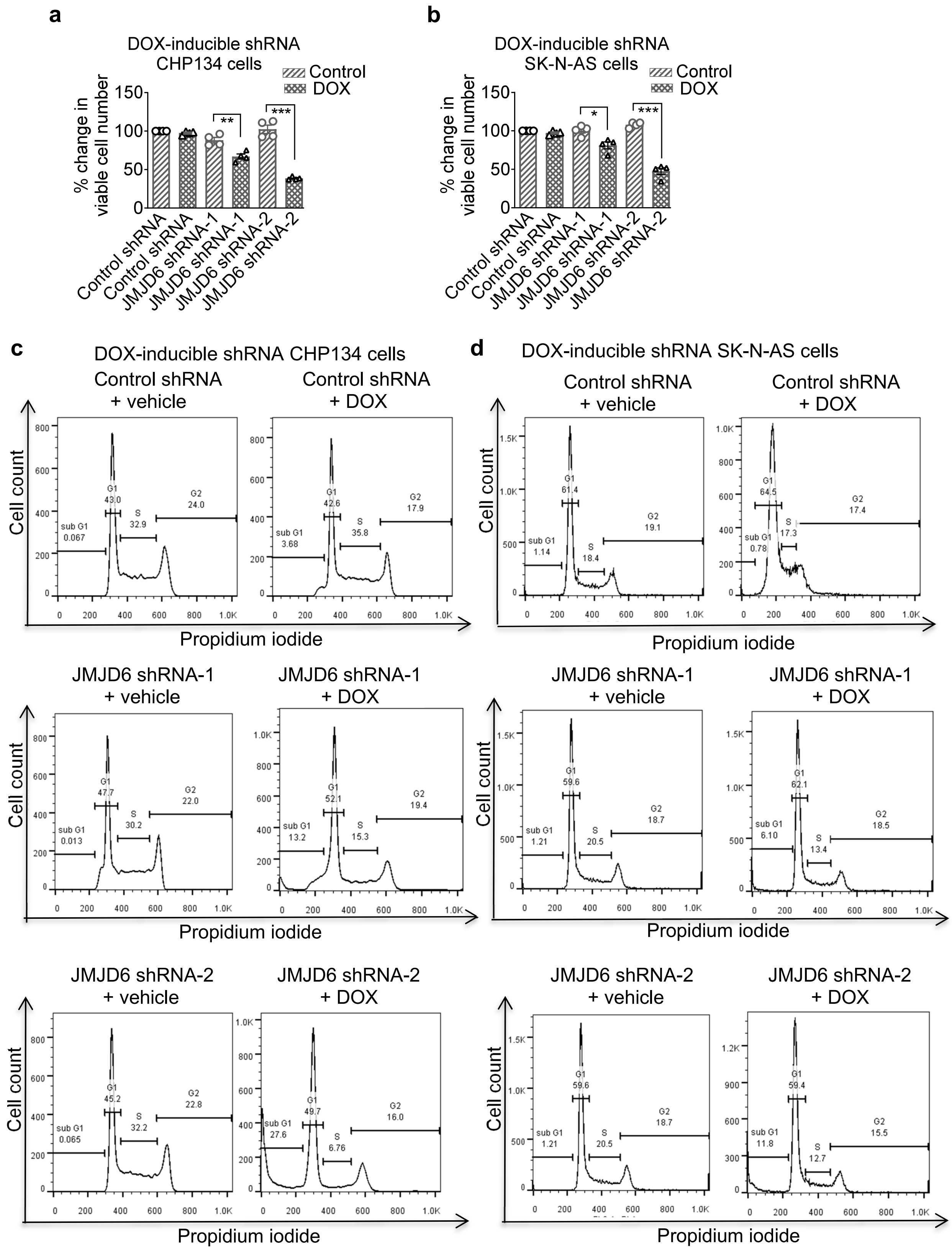
a
DOX-inducible shRNA
CHP134 cells
Control
DOX
% change in
viable cell number
150
100
50
0
**
***
Control shRNA
Control shRNA
JMJD6 shRNA-1
JMJD6 shRNA-1
JMJD6 shRNA-2
JMJD6 shRNA-2
b
DOX-inducible shRNA
SK-N-AS cells
Control
DOX
% change in
viable cell number
150
100
50
0
*
***
Control shRNA
Control shRNA
JMJD6 shRNA-1
JMJD6 shRNA-1
JMJD6 shRNA-2
JMJD6 shRNA-2
c
DOX-inducible shRNA CHP134 cells
Control shRNA
+ vehicle
Control shRNA
+ DOX
JMJD6 shRNA-1
+ vehicle
JMJD6 shRNA-1
+ DOX
JMJD6 shRNA-2
+ vehicle
JMJD6 shRNA-2
+ DOX
Cell count
Propidium iodide
d
DOX-inducible shRNA SK-N-AS cells
Control shRNA
+ vehicle
Control shRNA
+ DOX
JMJD6 shRNA-1
+ vehicle
JMJD6 shRNA-1
+ DOX
JMJD6 shRNA-2
+ vehicle
JMJD6 shRNA-2
+ DOX
Cell count
Propidium iodide

Supplementary Fig. 5. JMJD6 knockdown leads to neuroblastoma cell growth inhibition and apoptosis. **a, b** DOX-inducible control shRNA, JMJD6 shRNA-1 or JMJD6 shRNA-2 CHP134 (**a**) and SK-N-AS (**b**) cells were treated with vehicle control or 2μg/ml DOX for 96 hours, followed by Alamar blue assays. Error bars represent normalized standard errors from four independent experiments (* $p < 0.05$, ** $p < 0.01$, *** $p < 0.001$, two-tailed unpaired Student's t-test). **c, d** DOX-inducible control shRNA or JMJD6 shRNA-1 and JMJD6 shRNA-2 CHP134 (**c**) and SK-N-AS (**d**) cells were treated with vehicle control or DOX for 72 hours, followed by staining with propidium iodide and flow cytometry analysis of the cell cycle. The percentage of cells at each phase of the cell cycle was calculated. Source data are provided as a Source Data file.

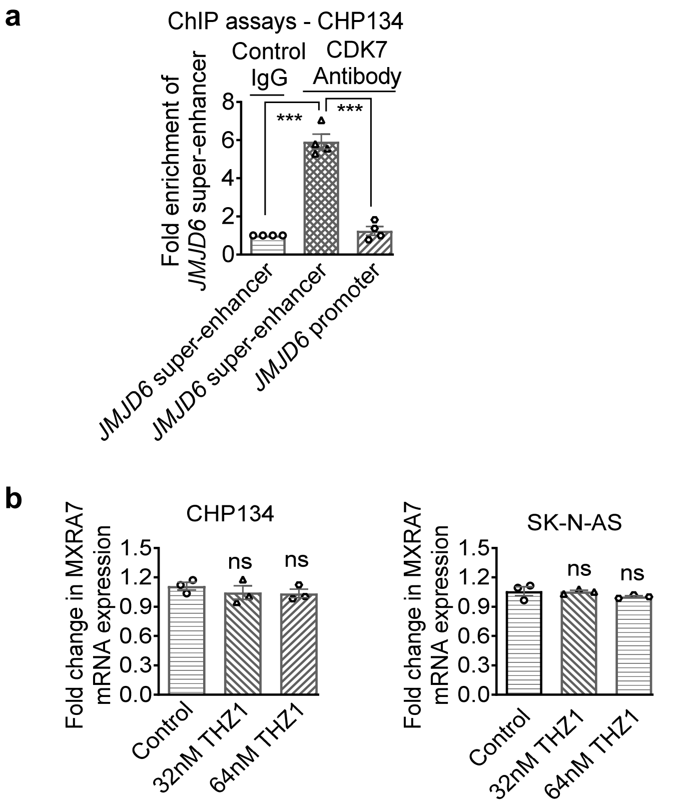

Supplementary Fig. 6. The super-enhancers at the *MXRA7*/*JMJD6* gene locus is bound by CDK7 but the CDK7 inhibitor THZ1 does not regulate *MXRA7* gene expression. **a** ChIP assays were performed with a control IgG or anti-CDK7 antibody, followed by PCR with primers targeting the *MXRA7*/*JMJD6* gene super-enhancer or *JMJD6* gene promoter, as shown in Fig. 6a, in CHP134 cells. Fold enrichment of the *JMJD6* gene super-enhancer or promoter regions was calculated as the difference in cycle thresholds obtained with the anti-CDK7 antibody compared with the control IgG, relative to input. Error bars represent normalized standard errors from four independent experiments (*** $p < 0.001$, two-tailed unpaired Student's t-test). **b** CHP134 and SK-N-AS cells were treated with vehicle control, 32 or 64 nM THZ1. RNA was extracted from the cells 24 hours later, and subjected to RT-PCR analysis of MXRA7 mRNA expression. Error bars represent normalized standard errors from three independent experiments (ns no significant difference). Source data are provided as a Source Data file.

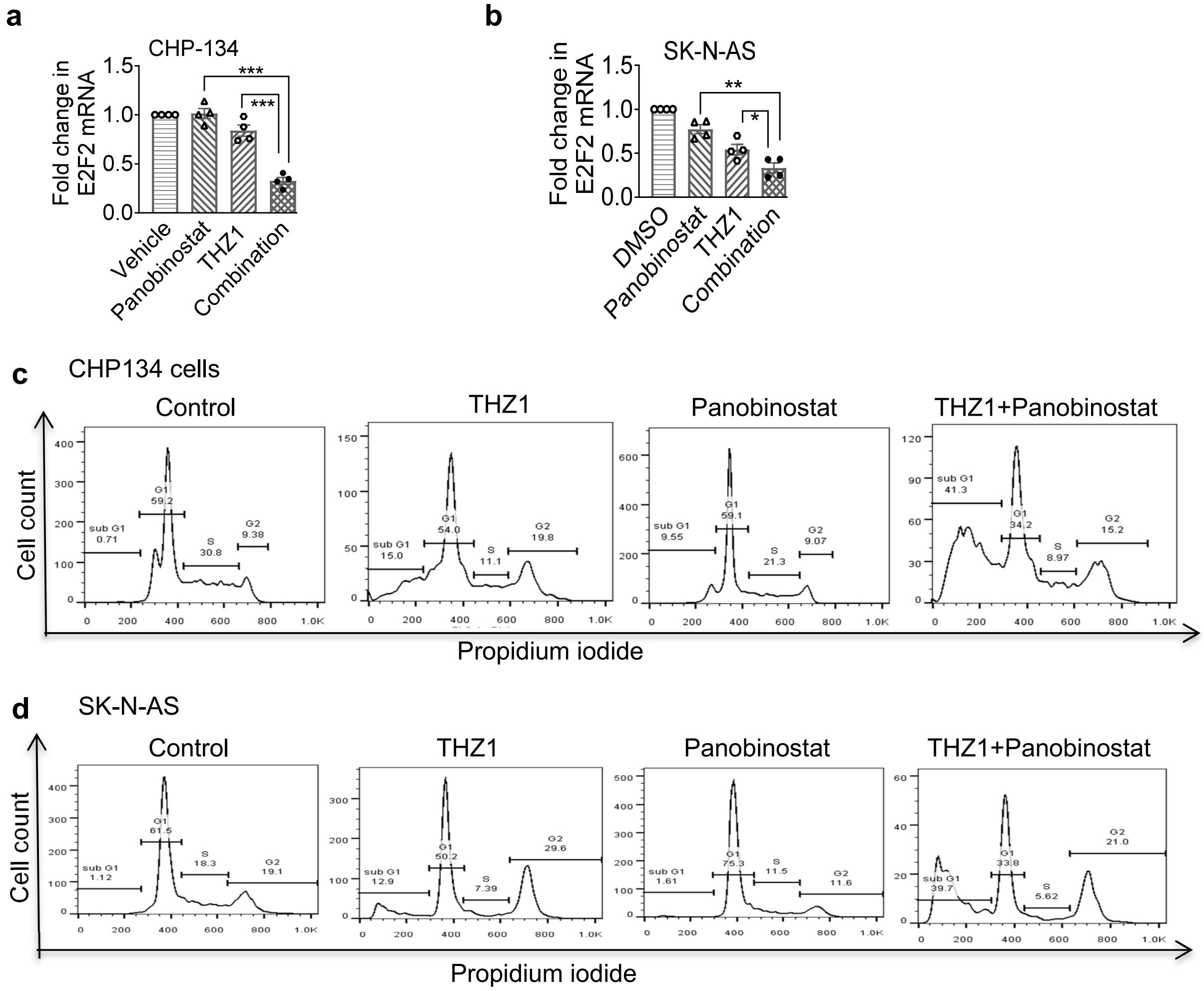

Supplementary Fig. 7. THZ1 and panobinostat synergistically reduce E2F2 expression and induce neuroblastoma cell apoptosis. **a, b** CHP134 (**a**) and SK-N-AS (**b**) cells were treated with vehicle control, 32nM THZ1, 10nM panobinostat, or combination of THZ1 and panobinostat for 24 hours. RT-PCR was performed to examine E2F2 mRNA expression. Error bars represent normalized standard errors from four independent experiments (* $p < 0.05$, ** $p < 0.01$, *** $p < 0.001$, two-tailed unpaired Student's t-test). **c, d** CHP134 (**c**) and SK-N-AS (**d**) cells were treated with vehicle control, 32nM THZ1, 10nM panobinostat, or combination of THZ1 and panobinostat for 72 hours, followed by staining with propidium iodide and flow cytometry analysis of the cell cycle. The percentage of cells at each phase of the cell cycle was calculated. Source data are provided as a Source Data file.

Supplementary Table 1. List of transcription factor target motifs at transcription start sites of genes regulated by JMJD6 shRNA-1 and JMJD6 shRNA-2 in CHP134 cells. Genome-wide differential gene expression studies were performed with microarray in DOX-inducible control shRNA, JMJD6 shRNA-1 and JMJD6 shRNA-2 CHP134 cells 40 hours after treatment with vehicle control or DOX. The microarray experiments were repeated four times. Gene set enrichment analysis generated enriched gene sets of transcription factor target motifs around transcription start sites of genes down-regulated by JMJD6 shRNA-1 and JMJD6 shRNA-2 by 1.5 fold with $p < 0.05$ and adjusted $p < 0.20$. Size represented the number of genes down-regulated by JMJD6 shRNA-1 and JMJD6 shRNA-2 in each gene set, ES and NES enrichment score and normalized enrichment score, and FDR false discovery rate. $p \leq 0.05$ and FDR ≤ 0.25 indicated significant events. miRNAs and unknown binding sites were not shown.

NAME	SIZE	ES	NES	NOM p value	FDR q-val	FWER p-val	RANK AT MAX
E2F1DP2_01	16	-0.38	-1.85	0.017892644	0.183653	0.589	395
E2F4DP2_01	16	-0.38	-1.83	0.008247423	0.13282	0.619	395
E2F1DP1_01	16	-0.38	-1.83	0.012422361	0.100113	0.622	395
E2F1_Q6	15	-0.38	-1.74	0.037328094	0.128669	0.791	395
E2F4DP1_01	15	-0.38	-1.71	0.024489796	0.122407	0.833	395

Supplementary Table 2. List of transcription factor target motifs at transcription start sites of genes down-regulated by JMJD6 shRNA-2 in SK-N-AS cells. Genome-wide differential gene expression studies were performed with microarray in DOX-inducible control shRNA and JMJD6 shRNA-2 SK-N-AS cells 40 hours after treatment with vehicle control or DOX. The microarray experiments were repeated for three times. Pre-ranked gene set enrichment analysis generated enriched gene sets of transcription factor target motifs around transcription start sites of genes down-regulated by JMJD6 shRNA-2. Size represented the number of genes down-regulated by JMJD6 shRNA-2 in each gene set, ES and NES enrichment score and normalized enrichment score, and FDR false discovery rate. P ≤ 0.05 and FDR ≤ 0.25 indicated significant events. miRNAs and unknown binding sites were not shown.

**NAME**	**SIZE**	**ES**	**NES**	**NOM p-val**	**FDR q-val**	**FWER p-val**	**RANK AT MAX**
E2F4DP2_01	17	-0.52	-2.213	0	0.11567	0.098	351
E2F1DP1_01	17	-0.52	-2.207	0.0031746	0.06007	0.102	351
SGCGSSAAA_E2F1DP2_01	15	-0.55	-2.176	0.0015244	0.05131	0.129	351
E2F1DP2_01	17	-0.52	-2.167	0	0.0404	0.135	351
E2F_02	17	-0.52	-2.166	0.001548	0.03253	0.136	351
E2F4DP1_01	19	-0.49	-2.143	0.0031797	0.03238	0.161	300
E2F1_Q6	18	-0.51	-2.123	0.0015798	0.03428	0.195	373
E2F1_Q6_01	15	-0.52	-2.071	0.0016447	0.04802	0.294	195
E2F_Q6	15	-0.49	-2.003	0.0031847	0.07158	0.437	351
E2F_Q4	15	-0.49	-1.904	0.0093313	0.12443	0.706	351
E2F1_Q4	18	-0.44	-1.878	0.0091603	0.13658	0.764	508
E2F_Q6_01	15	-0.47	-1.877	0.0047847	0.1266	0.764	373
E2F_03	20	-0.4	-1.815	0.0153139	0.17729	0.887	373

Supplementary Table 3. List of the top 20 gene sets with reduced RNA Pol II binding peaks at gene promoters after JMJD6 knockdown. ChIP sequencing experiments were performed with control IgG or anti-RNA Pol II antibody in DOX-inducible JMJD6 shRNA-2 CHP134 cells 40 hours after treatment with vehicle control or DOX. Genome-wide RNA Pol II binding peaks were identified and compared between control-treated and DOX-treated samples, and GSEA analysis was performed to identify gene sets with reduced Pol II binding at gene promoters after JMJD6 knockdown. Size represented the number of genes with reduced RNA Pol II binding at promoters after JMJD6 knockdown, and FDR false discovery rate. P ≤ 0.05 and FDR ≤ 0.05 indicated significant events.

HALLMARK Gene Set Name	Number of Genes in Gene Set (K)	Number of Genes in Overlap (k)	k/K	p-value
HALLMARK_G2M_CHECKPOINT	200	19	0.095	6.92E-14
HALLMARK_E2F_TARGETS	200	18	0.09	8.01E-13
HALLMARK_MITOTIC_SPINDLE	200	14	0.07	7.56E-09
HALLMARK_MYC_TARGETS_V1	200	11	0.055	3.31E-06
HALLMARK_HEDGEHOG_SIGNALING	36	5	0.139	2.06E-05
HALLMARK_IL2_STAT5_SIGNALING	200	10	0.05	2.11E-05
HALLMARK_MTORC1_SIGNALING	200	10	0.05	2.11E-05
HALLMARK_UV_RESPONSE_DN	144	8	0.056	6.73E-05
HALLMARK_HEME_METABOLISM	200	9	0.045	1.21E-04
HALLMARK_HYPOXIA	200	9	0.045	1.21E-04
HALLMARK_WNT_BETA_CATENIN_SIGNALING	42	4	0.095	6.33E-04
HALLMARK_MYC_TARGETS_V2	58	4	0.069	2.13E-03
HALLMARK_GLYCOLYSIS	200	7	0.035	2.88E-03
HALLMARK_FATTY_ACID_METABOLISM	158	6	0.038	3.82E-03
HALLMARK_UNFOLDED_PROTEIN_RESPONSE	113	5	0.044	4.26E-03
HALLMARK_CHOLESTEROL_HOMEOSTASIS	74	4	0.054	5.15E-03
HALLMARK_COMPLEMENT	200	6	0.03	1.16E-02
HALLMARK_OXIDATIVE_PHOSPHORYLATION	200	6	0.03	1.16E-02
HALLMARK_TNFA_SIGNALING_VIA_NFKB	200	6	0.03	1.16E-02
HALLMARK_UV_RESPONSE_UP	158	5	0.032	1.67E-02

FDR q-value	HALLMARK Gene Set Name
3.46E-12	HALLMARK_G2M_CHECKPOINT
2.00E-11	HALLMARK_E2F_TARGETS
1.26E-07	HALLMARK_MITOTIC_SPINDLE
4.14E-05	HALLMARK_MYC_TARGETS_V1
1.50E-04	HALLMARK_HEDGEHOG_SIGNALING
1.50E-04	HALLMARK_IL2_STAT5_SIGNALING
1.50E-04	HALLMARK_MTORC1_SIGNALING
4.21E-04	HALLMARK_UV_RESPONSE_DN
6.07E-04	HALLMARK_HEME_METABOLISM
6.07E-04	HALLMARK_HYPOXIA
2.88E-03	HALLMARK_WNT_BETA_CATENIN_SIGNALING
8.89E-03	HALLMARK_MYC_TARGETS_V2
1.11E-02	HALLMARK_GLYCOLYSIS
1.36E-02	HALLMARK_FATTY_ACID_METABOLISM
1.42E-02	HALLMARK_UNFOLDED_PROTEIN_RESPONSE
1.61E-02	HALLMARK_CHOLESTEROL_HOMEOSTASIS
3.05E-02	HALLMARK_COMPLEMENT
3.05E-02	HALLMARK_OXIDATIVE_PHOSPHORYLATION
3.05E-02	HALLMARK_TNFA_SIGNALING_VIA_NFKB
4.17E-02	HALLMARK_UV_RESPONSE_UP
