# Supplementary material for: JMJD6 is a tumorigenic factor and therapeutic target in neuroblastoma
Source: Nat Commun. 2019 Jul 25;10:3319. doi: 10.1038/s41467-019-11132-w (PMC6658504; doi:10.1038/s41467-019-11132-w)
Supplement: Supplementary file 3 — Description of Additional Supplementary Files [file 41467_2019_11132_MOESM3_ESM.pdf]

Description of Additional Supplementary Files

File Name: Supplementary Data 1.

Description: Genes up- or down-regulated by JMJD6 shRNA-1 and JMJD6 shRNA-2 compared with control shRNA, as identified by Affymetrix microarray analysis in DOX-inducible control shRNA, JMJD6 shRNA-1 and JMJD6 shRNA-2 CHP134 cells after treatment with vehicle control or DOX for 40 hours. The microarray experiments were repeated four times. Differential expression analysis was performed using the Limma package. Moderated t tests were performed with the Limma package. Genes with fold change of > 1.5, $p < 0.05$ and adjusted $p < 0.20$ were listed.

File Name: Supplementary Data 2.

Description: List of promoters with reduced RNA Pol II binding peaks after JMJD6 knockdown. ChIP sequencing experiments were performed with control IgG or anti-RNA Pol II antibody in DOX-inducible JMJD6 shRNA-2 CHP134 cells 40 hours after treatment with vehicle or DOX. Genome-wide RNA Pol II binding peaks were identified at genome assembly GRCh37 (hg19), expressed as normalised ChIP-sequencing reads per kilobase, and compared between control-treated and DOX-treated samples. The experiments were performed in duplicates. A paired Student's t-test of normalised ChIP-sequencing reads per kilobase across all the listed promoters was performed for replicates independently ($P < 0.0001$).

File Name: Supplementary Data 3.

Description: Promoter regions bound by JMJD6, by N-Myc or by both JMJD6 and N-Myc. Supplementary Data 3 is composed of 3 sub-data, 3a-c.
Supplementary Data 3a. Promoter regions bound by both JMJD6 and N-Myc. ChIP-Seq experiments were performed in triplicates with anti-JMJD6 or anti-N-Myc antibody in CHP134 cells. Genome-wide JMJD6 and N-Myc binding peaks were identified at genome assembly GRCh37 (hg19), and compared between triplicate JMJD6 antibody ChIP-Seq data and triplicate N-Myc antibody ChIP-Seq data. Only peaks with false discovery rate q-value < 0.05 were retained for analysis, and only promoter regions overlapping with peaks in all three replicates of N-Myc and JMJD6 antibody ChIP-Seq were listed.
Supplementary Data 3b. Promoter regions bound by JMJD6 protein. Anti-JMJD6 antibody ChIP-Seq experiments were performed in triplicates in CHP134 cells. Genome-wide JMJD6 binding peaks were identified at genome assembly GRCh37 (hg19), and compared among triplicate JMJD6 antibody ChIP-Seq data. Only peaks with false discovery rate q-value < 0.05 were retained for analysis, and only promoter regions overlapping with peaks in all three replicates of JMJD6 antibody ChIP-Seq were listed.
Supplementary Data 3c. Promoter regions bound by N-Myc protein. N-Myc antibody ChIP-Seq experiments were performed in triplicates in CHP134 cells. Genome-wide N-Myc binding peaks were identified at genome assembly GRCh37 (hg19), and compared among triplicate N-Myc antibody ChIP-Seq data. Only peaks with false discovery rate q-value < 0.05 were retained for analysis, and only promoter regions overlapping with peaks in all three replicates of N-Myc antibody ChIP-Seq were listed.

File Name: Supplementary Data 4.

Description: Typical enhancer regions bound by JMJD6, by N-Myc or by both JMJD6 and N-Myc. Supplementary Data 4 is composed of 3 sub-data, 4a-c.
Supplementary Data 4a. Typical enhancer regions bound by both JMJD6 and N-Myc. ChIP-Seq experiments were performed in triplicates with anti-JMJD6 or anti-N-Myc antibody in CHP134 cells. Genome-wide JMJD6 and N-Myc binding peaks were identified at genome assembly GRCh37 (hg19), and compared between triplicate JMJD6 antibody ChIP-Seq data and triplicate N-Myc antibody ChIP-Seq data. Only peaks with false discovery rate q-value < 0.05 were retained for analysis, and only typical enhancer regions overlapping with peaks in all three replicates of N-Myc and JMJD6 antibody ChIP-Seq were listed.
Supplementary Data 4b. Typical enhancer regions bound by JMJD6 protein. Anti-JMJD6 antibody ChIP-Seq experiments were performed in triplicates in CHP134 cells. Genome-wide JMJD6 binding peaks were identified at genome assembly GRCh37 (hg19), and compared among triplicate JMJD6 antibody ChIP-Seq data. Only peaks with false discovery rate q-value < 0.05 were retained for analysis, and only typical enhancer regions overlapping with peaks in all three replicates of JMJD6 antibody ChIP-Seq were listed.
Supplementary Data 4c. Typical enhancer regions bound by N-Myc protein. N-Myc antibody ChIP-Seq experiments were performed in triplicates in CHP134 cells. Genome-wide N-Myc binding peaks were identified at genome assembly GRCh37 (hg19), and compared among triplicate N-Myc antibody ChIP-Seq data. Only peaks with false discovery rate q-value < 0.05 were retained for analysis, and only typical enhancer regions overlapping with peaks in all three replicates of N-Myc antibody ChIP-Seq were listed.

File Name: Supplementary Data 5.

Description: Super-enhancer regions bound by JMJD6, by N-Myc or by both JMJD6 and N-Myc. Supplementary Data 5 is composed of 3 sub-data, 5a-c.
Supplementary Data 5a. Super-enhancer regions bound by both JMJD6 and N-Myc. ChIP-Seq experiments were performed in triplicates with anti-JMJD6 or anti-N-Myc antibody in CHP134 cells. Genome-wide JMJD6 and N-Myc binding peaks were identified at genome assembly

GRCh37 (hg19), and compared between triplicate JMJD6 antibody ChIP-Seq data and triplicate N-Myc antibody ChIP-Seq data. Only peaks with false discovery rate q-value < 0.05 were retained for analysis, and only super-enhancer regions overlapping with peaks in all three replicates of N-Myc and JMJD6 antibody ChIP-Seq were listed.

Supplementary Data 5b. Super-enhancer regions bound by JMJD6 protein. Anti-JMJD6 antibody ChIP-Seq experiments were performed in triplicates in CHP134 cells. Genome-wide JMJD6 binding peaks were identified at genome assembly GRCh37 (hg19), and compared among triplicate JMJD6 antibody ChIP-Seq data. Only peaks with false discovery rate q-value < 0.05 were retained for analysis, and only super-enhancer regions overlapping with peaks in all three replicates of JMJD6 antibody ChIP-Seq were listed.

Supplementary Data 5c. Super-enhancer regions bound by N-Myc protein. N-Myc antibody ChIP-Seq experiments were performed in triplicates in CHP134 cells. Genome-wide N-Myc binding peaks were identified at genome assembly GRCh37 (hg19), and compared among triplicate N-Myc antibody ChIP-Seq data. Only peaks with false discovery rate q-value < 0.05 were retained for analysis, and only super-enhancer regions overlapping with peaks in all three replicates of N-Myc antibody ChIP-Seq were listed.
