# Supplementary material for: JMJD6 is a tumorigenic factor and therapeutic target in neuroblastoma
Source: Nat Commun. 2019 Jul 25;10:3319. doi: 10.1038/s41467-019-11132-w (PMC6658504; doi:10.1038/s41467-019-11132-w)
Supplement: Supplementary file 9 — Reporting Summary [file 41467_2019_11132_MOESM9_ESM.pdf]

nature research

Corresponding author(s): Tao Liu

Last updated by author(s): Jun 11, 2019

Reporting Summary

Nature Research wishes to improve the reproducibility of the work that we publish. This form provides structure for consistency and transparency in reporting. For further information on Nature Research policies, see Authors & Referees and the Editorial Policy Checklist.

Statistics

For all statistical analyses, confirm that the following items are present in the figure legend, table legend, main text, or Methods section.

n/a	Confirmed	
☐	☒	The exact sample size (*n*) for each experimental group/condition, given as a discrete number and unit of measurement
☐	☒	A statement on whether measurements were taken from distinct samples or whether the same sample was measured repeatedly
☐	☒	The statistical test(s) used AND whether they are one- or two-sided<br>*Only common tests should be described solely by name; describe more complex techniques in the Methods section.*
☒	☐	A description of all covariates tested
☒	☐	A description of any assumptions or corrections, such as tests of normality and adjustment for multiple comparisons
☐	☒	A full description of the statistical parameters including central tendency (e.g. means) or other basic estimates (e.g. regression coefficient) AND variation (e.g. standard deviation) or associated estimates of uncertainty (e.g. confidence intervals)
☐	☒	For null hypothesis testing, the test statistic (e.g. *F*, *t*, *r*) with confidence intervals, effect sizes, degrees of freedom and *P* value noted<br>*Give P values as exact values whenever suitable.*
☒	☐	For Bayesian analysis, information on the choice of priors and Markov chain Monte Carlo settings
☐	☒	For hierarchical and complex designs, identification of the appropriate level for tests and full reporting of outcomes
☐	☒	Estimates of effect sizes (e.g. Cohen's *d*, Pearson's *r*), indicating how they were calculated

Our web collection on statistics for biologists contains articles on many of the points above.

Software and code

Policy information about availability of computer code

Data collection	Data collection in RT-PCR experiments used SDS Software v2.4. Flow cytometry results were collected using BD FACS Diva software. Cell viability assay results were collected using Wallace 1420 workstation. No custom algorithms or software was used.
Data analysis	For statistical analysis, experiments were performed at least 3 times with data analysed using Graphpad Prism 6.<br>Microarray data were analyzed in R (http://www.r-project.org/) with bioconductor packages (http://www.bioconductor.org/) and normalized with the Robust MultiArray Average algorithm, and differential expression analysis was performed using the Limma package3. Moderated t-tests were performed with the Limma package and the false discovery rate (FDR) was controlled using the Benjamini and Hochberg method. Functional enrichment of the differentially expressed genes was performed with Gene Set Enrichment Analysis (GSEA). The list of differentially expressed genes were ranked by the log of the fold change and then compared to known gene sets in the curated database Molecular Signature Database. No custom algorithms or software was used.

For manuscripts utilizing custom algorithms or software that are central to the research but not yet described in published literature, software must be made available to editors/reviewers. We strongly encourage code deposition in a community repository (e.g. GitHub). See the Nature Research guidelines for submitting code & software for further information.

Data

Policy information about availability of data

All manuscripts must include a data availability statement. This statement should provide the following information, where applicable:

- Accession codes, unique identifiers, or web links for publicly available datasets
- A list of figures that have associated raw data
- A description of any restrictions on data availability

The microarray data and the ChIP-Seq data have been
deposited at the Gene Expression Omnibus website with series numbers of GSE112914,
GSE113139 and GSE112919 respectively. All relevant data are available from the authors.

Field-specific reporting

Please select the one below that is the best fit for your research. If you are not sure, read the appropriate sections before making your selection.

☒ Life sciences ☐ Behavioural & social sciences ☐ Ecological, evolutionary & environmental sciences

For a reference copy of the document with all sections, see nature.com/documents/nr-reporting-summary-flat.pdf

Life sciences study design

All studies must disclose on these points even when the disclosure is negative.

Sample size	All in vitro experiments were performed at least three times and were based on previous experience with each experimental setup. The sample size is acceptable in the field.<br>Animal sample sizes were estimated according to our previous work (Liu PY et al. J Natl Cancer Inst 106, pii: dju113 (2014)) in which we carried out similar studies in neuroblastoma progression.
Data exclusions	Animals were only excluded from analyses when they were excluded from the experiments, because they did not develop tumors after xenografting with neuroblastoma cells.
Replication	All experiments were repeated at least three times. Mouse experiments were performed in at least 10 mice per experimental group. All attempts to replicate the data were successful.
Randomization	For in vivo xenograft experiments, animals where randomly allocated to experimental treatment groups as tumour size reached the treatment start point of 0.05cm3.
Blinding	No blinding was performed in animal experiments, because the dox feed treatment was given by cage. For analysing gene expression in human tumor tissues, the investigators were blinded during analyses.

Reporting for specific materials, systems and methods

We require information from authors about some types of materials, experimental systems and methods used in many studies. Here, indicate whether each material, system or method listed is relevant to your study. If you are not sure if a list item applies to your research, read the appropriate section before selecting a response.

Materials & experimental systems

n/a	Involved in the study	
☐	☒	Antibodies
☐	☒	Eukaryotic cell lines
☒	☐	Palaeontology
☐	☒	Animals and other organisms
☒	☐	Human research participants
☐	☒	Clinical data

Methods

n/a	Involved in the study	
☐	☒	ChIP-seq
☐	☒	Flow cytometry
☒	☐	MRI-based neuroimaging

Antibodies

Antibodies used	Monoclonal mouse ANTI-FLAG M2 antibody (F3165, Sigma)<br>Rabbit anti-BRD4 antibody (A301-985A100 Bethyl Laboratories Montgomery, TX)<br>Rabbit anti-JMJD6 antibody (Abcam, ab64575)<br>Mouse anti-RNA Pol II antibody (664903, BioLegend, San Diego, CA)<br>Rabbit anti-H3K27ac antibody (ab4726, Abcam)<br>Rabbit anti-H3K4me antibody (ab106165, Abcam)<br>Rabbit anti-H3K4me3 antibody (ab213224, Abcam)<br>Mouse anti-N-Myc (sc-53993, Santa Cruz Biotechnology)<br>Rabbit anti-c-Myc (ab56, Abcam)<br>Mouse anti-CDK7 (sc-7344, Santa Cruz Biotechnology)<br>Mouse anti-E2F2 antibody (sc-633X, Santa Cruz Biotechnology)<br>Mouse anti-JMJD6 antibody (sc-28348, Santa Cruz Biotechnology)<br>Mouse and rabbit anti-c-Myc antibody (1:500, Santa Cruz Biotechnology).
Validation	All antibodies were validated previously by the manufacturers according to their datasheet and/or according to published papers. We also validated all the antibodies upon delivery for each experimental method.

Eukaryotic cell lines

Policy information about cell lines

Cell line source(s)	HEK 293T cells were obtained from the American Type Culture Collection in 1998. CHP134 and SK-N-AS cells were obtained from the European Collection of Cell Cultures and Sigma Aldrich in 2010.
Authentication	Cell line identity was verified in 2014, 2015, 2016 and 2017 by small tandem repeat profiling conducted at the Garvan Institute of Medical Research or Cellbank Australia.
Mycoplasma contamination	All cell lines were confirmed to be mycoplasma free by quarterly mycoplasma testing using the MycoAlert™ mycoplasma detection kit (Lonza).
Commonly misidentified lines (See ICLAC register)	No commonly misidentified cell lines were used.

Animals and other organisms

Policy information about studies involving animals; ARRIVE guidelines recommended for reporting animal research

Laboratory animals	Female Balb/c nude mice aged 5 to 6 weeks were used in this JMJD6 shRNA xenograft study.<br>For the experimental therapy study, male Balb/c nude mice aged 5 to 6 weeks were used.
Wild animals	None
Field-collected samples	None
Ethics oversight	This mouse experiment was approved by the Animal Care and Ethics Committee of UNSW Sydney, Australia, and animals' care was performed in agreement with institutional guidelines.<br>The experiment was approved by the Shanghai University of Traditional Chinese Medicine Committee on the Use of Live Animals for Teaching and Research, and animals' care was performed in accordance with the Guide for the Care and Use of Laboratory Animals published by the National Institutes of Health (publication No. SCXX(HU) 2007-0005).

Note that full information on the approval of the study protocol must also be provided in the manuscript.

Clinical data

Policy information about clinical studies

All manuscripts should comply with the ICMJE guidelines for publication of clinical research and a completed CONSORT checklist must be included with all submissions.

Clinical trial registration	*Provide the trial registration number from ClinicalTrials.gov or an equivalent agency.*
Study protocol	*Note where the full trial protocol can be accessed OR if not available, explain why.*
Data collection	*Describe the settings and locales of data collection, noting the time periods of recruitment and data collection.*
Outcomes	*Describe how you pre-defined primary and secondary outcome measures and how you assessed these measures.*

ChIP-seq

Data deposition

☒ Confirm that both raw and final processed data have been deposited in a public database such as GEO.

☒ Confirm that you have deposited or provided access to graph files (e.g. BED files) for the called peaks.

Data access links<br>*May remain private before publication.*	Super series title: JMJD6 gene gain is a tumorigenic factor and therapeutic target in neuroblastoma<br>Series GSE113140<br>https://www.ncbi.nlm.nih.gov/geo/query/acc.cgi?acc=GSE113140
Files in database submission	GSM3097929 h3k4me_WON3029<br>GSM3097930 h3k4me3_WON3029<br>GSM3097931 h3k27ac_WON3029<br>GSM3097932 input_WON3029<br>GSM3097933 rb IgG_WON3029<br><br>GSM3490535 Sample_Input_DOX_WON5767<br>GSM3490536 Sample_Input_NT_WON5767<br>GSM3490537 Sample_pol2_DOX_WON5767

GSM3490538 Sample_pol2_NT_WON5767
GSM3490539 Sample_Input_DOX
GSM3490540 Sample_Input_NT
GSM3490541 Sample_pol2_DOX
GSM3490542 Sample_pol2_NT

Genome browser session (e.g. UCSC)

Revised version:
http://genome.ucsc.edu/s/Jasewong/GEO%20submission%3A%20Series%20GSE113140

Methodology

Replicates

The ChIP-SEQ of histone marks: H3K27ac, H3K4me and H3K4me3 was done once.
The anti-RNA Pol II antibody pulldown in DOX-inducible JMJD6 shRNA-2 CHP134 cells, treated with vehicle control or DOX was done in duplicate.
The JMJD6 and NMYC pulldowns in CHP134 cells was done in triplicate.

Sequencing depth

Each sample had a minimum of 15 million reads, using single-end reading, at 75 bp in size.

Antibodies

Rabbit anti-H3K27ac antibody (ab4726, Abcam), rabbit anti-H3K4me antibody (ab106165, Abcam), rabbit anti-H3K4me3 antibody (ab213224, Abcam), or control rabbit IgG (sc-2027, Santa Cruz Biotech).
Mouse anti-RNA Pol II antibody (664903, BioLegend) or control mouse IgG (sc-2025, Santa Cruz Biotech)

Peak calling parameters

Peaks were called with MACS2 'callpeak' (version macs2 2.1.1.20160309) against input controls, with default parameters.

Data quality

Pol2 control treatment and DOX treatment JMJD6 shRNA-2 CHP134 cells ChIP-seq was done in duplicate. To select genes with reduced RNA Pol II binding at their promoters after DOX treatment in DOX-inducible JMJD6 shRNA-2 CHP134 cells, gene promoters were selected as ± 1 kilobase from the transcription start site of genes from the RefSeq (refFlat) database, downloaded from the University of California Santa Cruz (UCSC) Table Browser. Promoters with reduced RNA Pol II binding were selected as those that had a RNA Pol II ChIP-Seq peak called within the region in both vehicle control-treated replicate samples, but no peak in either DOX-treated replicate samples. The statistical difference between normalized numbers of RNA Pol II ChIP-Seq reads between vehicle control-treated samples and DOX-treated samples was determined separately for each replicate using a paired t-test. The data points used to calculate statistical significance were "normalized ChIP-Seq reads per kilobase", determined using the following formula: total number of reads overlapping region of interest / size of region of interest (base pairs) / normalization factor * 1000, where normalization factor was equal to the number of ChIP-seq reads in the file divided by the average number of ChIP-seq reads across all files.
Enhancer and superenhancer regions were identified using H3K27ac data as described by Jiang et al (Nucleic Acids Research, Volume 47, Issue D1, 08 January 2019, Pages D235–D243). For enhancer and superenhancer gene associations, each gene was assigned a regulatory domain that extends ± 1000 kb in both directions to the nearest gene's transcription start site using the Genomic Regions Enrichment of Annotations Tool (GREAT). The two nearest gene regions overlapping with a given enhancer or superenhancer are listed alongside each region. Promoters, enhancers and superenhancers bound by NMYC or JMJD6 were selected if they overlapped with a ChIP-seq peak identified from all three replicates of either NMYC or JMJD6.

Software

ChIP-Seq raw reads were aligned using the Burrows Wheeler Aligner (BWA) with default parameters. Files were further processed using SAMtools, BEDtools and bedGraphToBigWig.

Flow Cytometry

Plots

Confirm that:

☒ The axis labels state the marker and fluorochrome used (e.g. CD4-FITC).

☒ The axis scales are clearly visible. Include numbers along axes only for bottom left plot of group (a 'group' is an analysis of identical markers).

☒ All plots are contour plots with outliers or pseudocolor plots.

☒ A numerical value for number of cells or percentage (with statistics) is provided.

Methodology

Sample preparation

Neuroblastoma cells were treated with vehicle control, THZ1, panobinostat, or combination of THZ1 and panobinostat for 72 hours. In separate experiments, DOX-inducible control shRNA, JMJD6 shRNA-1 or JMJD6 shRNA-2 neuroblastoma cells were treated with vehicle control or DOX for 72 hours. Cells were then collected and resuspended in solution containing 2µg/ml RNase (Sigma) and 50µg/ml propidium iodide at 2×106 cells/ml (Sigma).

Instrument

The FACS Calibur (BD Biosciences) machine was used for cell sorting.

Software

FACS Diva software (BD Biosciences) was used to collect data.

Cell population abundance	The percentage of cells in each stage of the cell cycle is detailed in Supplementary figures 5b, 5c, 7c and 7d.
Gating strategy	For analysis, neuroblastoma cells were first gated by SSC and FSC to seperate single cells from doublet cells. Cell debris was gated out and the percentage of cells in each cell cycle phase was quantified using FlowJo_V10, by fitting Gaussian curves to each sample.

☒ Tick this box to confirm that a figure exemplifying the gating strategy is provided in the Supplementary Information.
